# Analysis of gunshot damage to the porcine femur in a human thigh model using 5.5 mm airgun pellet: 3D reconstruction of gunshot injuries

**DOI:** 10.1371/journal.pone.0328767

**Published:** 2025-07-18

**Authors:** Mateusz Wilk, Małgorzata Chowaniec, Elżbieta Chowaniec, Grzegorz Bajor

**Affiliations:** 1 Collegium Medicum, WSB University, Dąbrowa Górnicza, Poland; 2 Department of Anatomy, Medical Faculty in Katowice, Medical University of Silesia, Katowice, Poland; 3 GC Adwokaci Gradowska Chowaniec Partnership, Czeladź, Poland; University of Perugia: Universita degli Studi di Perugia, ITALY

## Abstract

The performance of advanced air rifles available on the market is comparable to that of small-calibre firearms. The airgun market offers airgun pellets that vary in weight, shape, calibre and material. In view of the ease of airguns tuning, a study of the gunshot damage to the anterior surface of the porcine femoral shaft was carried out with shots fired from a 5.5 mm calibre air rifle. An original human thigh model using a pig femur embedded in ballistic gellatine was used in the study. Gunshot damage was inflicted by firing Haendler&Natterman Baracuda, HollowPoint, Spitzkugel and Excite Apollo 5.5 mm airgun pellets from an Air Arms s410 Hi-Power Xtra FAC 5.5 mm calibre PCP air rifle. Measurements of the velocity and impact energy of the pellets as well as the extent of the bone and periosteal entry damage were taken. Statistical analysis was used to identify differences between pellets with regard to dimensions of gunshot damage to the shafts of the femur. Selected models were subjected to CT imaging.

## Introduction

Many types of pneumatic devices can be found on the militaria market. The development of this shooting branch has evolved considerably in the last twenty years, resulting in the emergence of high-tech air rifles and air pistols with significantly higher discharge energies. The discharge energies generated by these pneumatic devices are bringing them ever closer to the ballistic performance hitherto presented by firearms and ammunition using gunpowder propellant. The airgun ammunition market has also expanded considerably, offering users access to extremely varied airgun pellets in terms of calibre, shape, and material. Gunshot injuries depend on the characteristics of the projectiles, the impact energy, and the anatomical area struck by the projectile. These different characteristics can be expected to result in a range of severity of injuries. For this reason, and because it is relatively easy it is relatively easy to “tune” airguns commonly available on the market, it was decided to start the study by assessing the penetration damage to the anterior femoral surface with gunshots from 5.5 mm calibre airguns.

### Crimes committed in Poland with air weapons

In Polish literature of the subject, the terms ‘pneumatic weapon’ and ‘airgun’ are used interchangeably. This is not an accurate statement, as every pneumatic weapon is an airgun, while not every airgun is currently legally classified as a pneumatic weapon. According to Article 8 of the Weapons and Ammunition Act of 21 May 1999 (Journal of Laws 1999 No 53, item 549), a pneumatic weapon is defined as “a device dangerous to life or health, which as a result of the action of compressed gas is capable of discharging a projectile from a barrel or a component replacing it and thus capable of striking a target at a distance, with the kinetic energy of the projectile leaving the barrel - or the component replacing it - exceeding 17 J” [1]. The data from police statistics indicate that fewer crimes have been committed each year with the use of broad-ranging air weapons in comparison with crimes involving firearms. It should be noted, however, that the police statistics do not distinguish between air devices with kinetic energy < 17J energy and air weapons with kinetic energy > 17J. Due to the obligation of registration of air weapons at the police, the number of crimes involving legally possessed air weapons is probably insignificant. However, the ease of modification of airguns with kinetic energy below 17J poses a risk of an easy access to illegal possession of pneumatic weapons of significant energy. These cases remain without any legal or administrative oversight.

**Fig 1:**
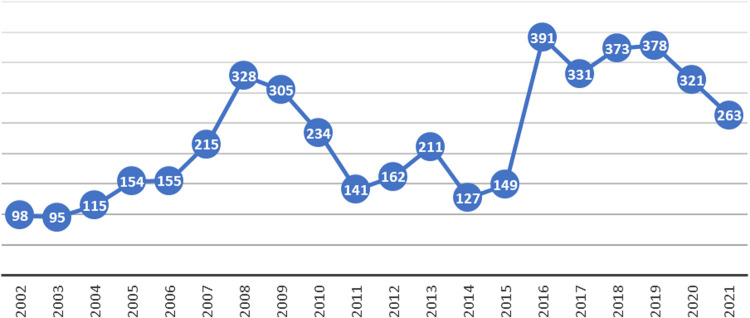
Number of crimes committed with broad-ranging air weapons between 2002 and 2021.

In Fig 1, a slow upward trend in the number of crimes committed with air weapons in Poland can be observed, with two crime peaks – in 2008–2009 and 2016–2019 [2]. These are the official data, although the statistics are most likely underestimated by about 50% [3].

The above factors and the lack of data in the scientific literature, both domestic and international, were the reasons for undertaking research using a human thigh model to assess gunshot injuries from pneumatic weapons.

### Long bone injuries from firearms and air weapons in the available literature

During combat operations in Iraq and Afghanistan between 2005 and 2009, 370 lower limb bone fractures inflicted by gunshot injuries were reported among US soldiers. Out of those, 141 involved femurs (38% of all gunshot fractures of lower limb bones) [4]. The minimum impact velocity of a bullet to penetrate bone is assumed to be at least 60–170 m/s, with the actual extent of injury and the depth of penetration of the bullet into the tissues depending on a number of factors, e.g, the anatomical area, the type and mass of the bullet, but also the type of clothing (uniforms). [5].

Few case reports of air weapons injuries have been presented in the gunshot literature. Tissue damage, including bone damage, inflicted by firearms [6] and organ damage (eyeball, scapula, flank flaps, liver) inflicted by pneumatic devices that do not meet the criteria of an air weapon, due to insufficiently low shot energy, have been very well described [7]. In the past, studies of femoral shafts airgun injuries with high impact energy projectiles have been conducted [8–10] however, in these studies, chromium steel bullets of 0.25 inch (6.35 mm) and 0.406 inch (10.03 mm) in diameter were fired into fixed human femurs not coated with ballistic gelatine. Therefore, the results referred only to the behaviour of an isolated femur in contact with the projectile without the impact of soft tissues on the extent of damage. In 2010, a study was performed on the airgun pellet damage to long bones; however, its limitations involve the use of airguns with a power of only 15.3 J and shots performed at fragments of bovine femoral shaft embedded in ballistic gelatine [11]. In 2018, an article was published that described a single shot of the porcine femur with a so-called FSP (fragment simulating projectile), with reported penetration of a single bone layer without going all the way through the shaft [12]. A little later, a paper was published showing the correlation between FSP impact energy and the degree of damage to the ovine fibula, simulating the fibula of a 5-year-old child. [13] The model used bones that were not surrounded by ballistic gelatine. It was therefore not possible to assess the movement of bone surrounded by soft tissues. There are also descriptions of case studies regarding airgun-related injuries, mostly focusing on head injuries. In one of these, Glowiński et al. [14] described a case of mortal head injury inflicted by a 5.50-mm caliber pellet fired from an air gun. The victim was shot with diabolo-type pellet fired from Kandar B3-3 spring air gun, with muzzle kinetic energy < 17J. A gunshot wound on the head was 5 mm in diameter and located in the left temporal-epidural region. The victim died due to intracranial hemorrhage. This case shows, that even air guns available without registration may cause a serious threat and inflict serious organ damage and even death of the human. Nonetheless, literature on the subject of airguns lacks an extensive description of gunshot injuries inflicted by air rifles.

### Study objectives

The study was undertaken to investigate how the type of airgun pellet and its impact energy affect gunshot damage to the anterior surface of the porcine femoral shaft located in a human thigh model. The secondary aim of the study was to create 3D reconstruction of airgun-related long bone damage.

## Materials and methods

### Description of the human thigh model used in the study

**Fig 2:**
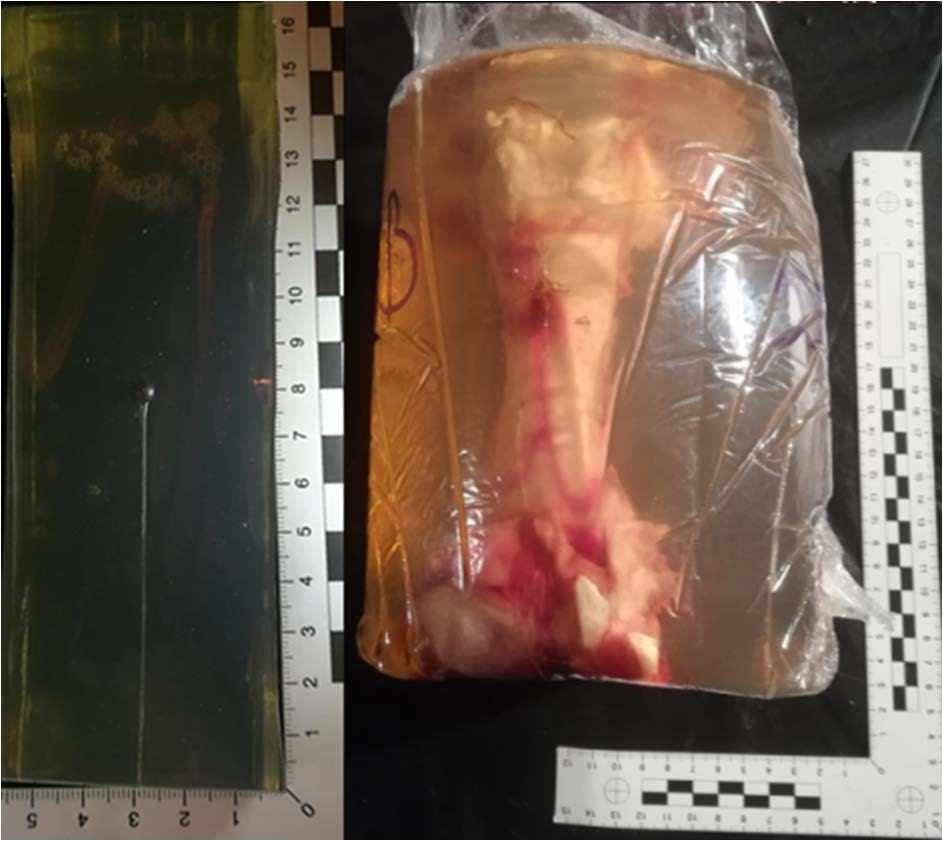
Result of an exemplar calibration test of ballistic gelatine (left), and a ready-made human thigh model (right).

The human thigh model featured a pork femur embedded in a cylinder of 10% Bloom 240 ballistic gelatine with an outer diameter of about 155 mm and a height of about 250 mm (Fig 2). The FBI model was chosen (concentration 10%, gelatine temperature 4^0^C) [15] due to better transparency of the ballistic gel compared to the US Army model (concentration 20%, gelatine temperature 10^0^C). A sample of the gelatine thus prepared was calibrated according to the protocol [16].

### Research stages

Before beginning of the studies, approval from Local Ethical Committee for Animal Experiments in Katowice was obtained (issue PCN/022/LKE/4/20)

The first stage of the research involved calibration of ballistic gelatine, for which the Crosman C2100 airgun and Razorgun pellet calibre 4.46 mm were used. Pellet impact velocities of approximately 163–166 m/s were obtained, yielding average penetration results in gelatine ranging from 73–79 mm according to the procedure [16] as shown in Fig 2. Shooting was conducted on an indoor shooting range under constant and unchanging temperature conditions of (+3°C to +5°C), humidity of 60%, artificial lighting and windless conditions.

**Fig 3:**
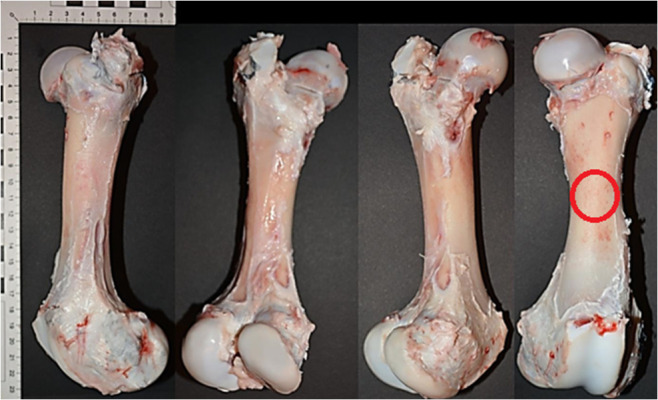
Fresh pork femurs, view from left: lateral, posterior, medial, anterior with the shot site marked with a red circle.

The second step was to analyse the characteristics of the pig femur (Fig 3). The pig femur is anatomically very similar to the human femur, however, it is significantly shorter. Its average length, calculated on the basis of measurements taken during the research, is about 240 mm and the diameter of the shaft in the A-P projection at the midpoint of its length averages about 30 mm. It should be noted that in terms of the diameter of the shaft, the pig femur is similar to the human femur [17].

Ten bones were measured. The bones were harvested from pigs of different sexes, weighing approximately 120 kg, which, given the shift of the centre of gravity among the pigs towards the rear, may indicate that the pressure on both hind limbs was approximately 65–70 kg, which is close to the body weight of a model human (70 kg).

The exact parameters of the bones used in the study can be found in Table 1.

**Table 1:** Parameters of the pig bones used in the study. The data are based on examination of 10 bones. (A-P: front-to-back, B-B: side-to-side, SD: standard deviation, Max: maximum value, Min: minimum value).

Parameter	Mean	SD	Max	Min
Overall length [mm]	239,12	5,48	265,10	220,91
Diameter of bone shaft A-P [mm]	30,63	1,62	35,77	26,39
Average cortical thickness of the anterior shaft of the bone [mm]	4,35	0,26	4,48	4,12
Weight of bone [g]	410,63	20,22	443,12	378,00

**Fig 4:**
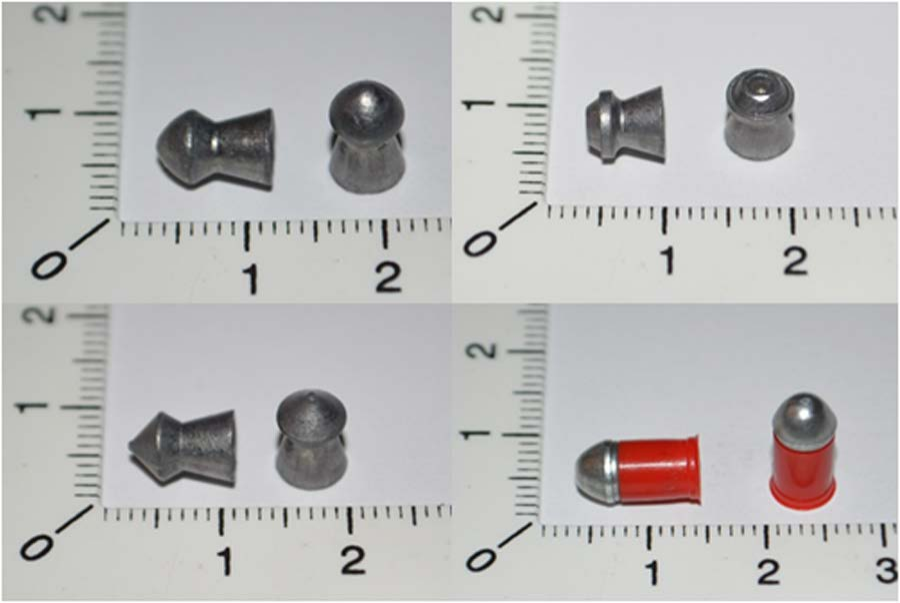
Airgun pellets used in the study. 1 – H&N Baracuda, 2 – H&N HollowPoint 3 – H&N Spitzkugel, 4 – H&N Excite Apollo.

The third stage required firing shots to measure the impact velocity and impact energy of the airgun pellets and to shoot human thigh models. Due to the character of models tested, which comprised of ballistic gelatine, all the test were performed in February and March 2024. The time of tests was planned so that the air temperature was between 3 and 8 degrees Celsius to avoid both freezing and excessive liquefaction of the models during transport to the firing range and to the CT laboratory. Test shots were fired from an Air Arms s410 Hi-Power Xtra FAC calibre 5.5 mm PCP air rifle, which allows shots with a maximum projectile energy of approximately 46J [18]. 5.5 mm calibre H&N Baracuda, HollowPoint, Spitzkugel and Apollo airgun pellets were used in the study (see Table 2, Fig 4). Shots were fired at a distance of 10 metres. This is the distance used for sport and recreational shooting. At this distance, the airgun pellets retain their stable ballistic properties and there is no significant drop in velocity, with the risk of ricochet directed towards the shooter reduced. Shots were fired centrally into the anterior surface at the midpoint of the femoral shaft. Models, where ricochets appeared, were excluded from further investigation.

**Table 2:** Airgun pellets used in the study.

Type of airgun pellet
H&N Baracuda cal. 5.5 mm – heavy airgun pellet, with conical, slightly rounded head. Very stable and accurate at long distances. Substantial impact force, moderate deformation on impact with deep penetration [19]. Weight 1,27g
H&N HollowPoint cal. 5.5 mm - with a characteristic depression in the central apical part of the head, resembling bursting ammunition with a tendency to deformation and limited penetration ability. [20]. Weight 0,82g
H&N Spitzkugel cal. 5.5 mm - pointed, moderate in weight. The shape of its head makes it a pellet with good penetrating properties, with reduced risk of ricochet in the when hitting a soft target. [21]. Weight 1,04g
H&N Excite Apollo cal. 5.5 mm consists of a metal alloy part and a Teflon collar surrounding, which serves as a seal in the barrel. It is characterized by very high piercing properties. It is made completely lead-free [22]. Weight 0,94g

**Fig 5:**
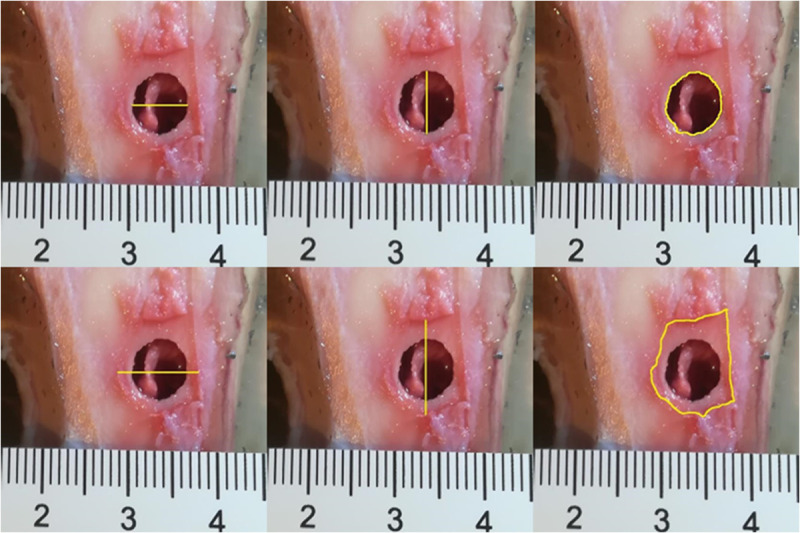
Entrance wound damage measurements based on photographic documentation.

The damage to selected models was imaged using a Carestream CS9600 CT scanner. Imaging was performed using a 150-micrometre thick layer and metal imaging artefact reduction software. Following the radioimaging, the models were sliced for macroscopic assessment of the gunshot damage. ImageJ software was used in the analysis of photographic data, taking the measurements shown in Fig 5. The RA ratio – the ratio of the area of entry damage in the periosteum to the area of entry damage in the bone – was also calculated.

### Statistical analysis

As part of the statistical analysis of the results of the study, the estimators of the positional parameters and the expected value and standard deviation were determined for all variables; in addition, the hypothesis of normality of distribution was verified using the Shapiro-Wilk test.

The verification of statistical hypotheses concerning the comparison of the samples analysed, in view of each positive verification of the normality of the distribution, was carried out using parametric tests: the test for two means preceded by the test for two variances, the test of homogeneity for many means preceded by the test for homogeneity of many variances (Bartlett’s test and Levene’s test). Depending on the results of the tests for homogeneity of multiple variances, the following were used:

- classic variant (one-way ANOVA) for cases of positively verified homogeneity of variance,
- variant with Brownian-Forsythe correction for cases of negatively verified homogeneity of variance.

For those cases, where the result of the classical variant of the analysis of variance indicated significant differences, Fisher’s LSD post-hoc tests were used. In cases to the contrary, Games-Howell post-hoc tests were used.

## Results

### Results of ballistic measurements

#### Airgun pellet damage to the anterior surface of the shaft of the porcine femur

Legend for the results: AB5 – H&N Baracuda cal. 5.5 mm, AH5 – H&N Hollow Point cal. 5.5 mm, AS5 – H&N Spitzkugel cal. 5.5 mm, AA5 – H&N Excite Apollo cal. 5.5 mm, Inlet – entrance wound.

## Graphic results

### Discussion

The literature reviewing the problem of gunshot injuries from high-powered pneumatic weapons, especially using human models, is rare. Published reports concern pneumatic devices with a discharge energy of less than 17J. Apart from a few papers, there is a lack of medico-legal studies on gunshot injuries to long bones inflicted by pneumatic weapons. Given the general descriptions of gunshot injuries involving pneumatic weapons in forensic textbooks available on the publishing market – e.g, by DiMaio [7] – the research undertaken was of a pioneering nature. In the literature on the subject, similar studies include work by T-T.N.Nguyen et al. [12], in which authors investigated the effect of so-called fragment simulating projectiles (FSPs) for the study of long bone fractures. In that research, in addition to proposing a research model, a single porcine femur gunshot test was performed, yet without describing the physical properties of the bone used for the test. A 4.5 mm calibre cylinder made of carbon steel weighing 0.78g was used as the fragment equivalent in the test. From the data obtained by this investigator, the impact energy in a single penetration test was approximately 41J. No bone shaft perforation was obtained, only partial penetration of the cortex occurred. The measurements of the gunshot injury area were not given either. The thigh model used 20% gelatine with a 300 Bloom factor, yet no dimensions, in particular the width of the model that was made, were given. With regard to this study, it is difficult to compare the results presented in this paper to those obtained by T-T.N.Nguyen. The authors of that study used a similar but non-identical test model for single trials. Notably, the projectiles fired were cylindrical 4.5 mm calibre pellets, constructed from carbon steel, which is much harder than the lead pellets used in the manufacturing of airgun pellets. In addition, presumably due to the test conditions, 20% Bloom 300 gelatine presumably at 10 degrees C was used – as pointed out in the article – obtaining a much more dense medium compared to the 10% Bloom 240 gelatine at 4 degrees C used in our study. The lack of bullet penetration at such high impact energy in the test model may have been related to the much higher density of the gelatine used in the tests and the resulting significantly higher resistance that the ballistic gelatine presented to the cylindrical projectile compared to the airgun shot. The difference in gelatin density and lack of dimensional information prevents direct comparison of the results with the present study.

In another paper by the same author [13], the fibula of a sheep was used as a test model, which, intended to simulate fibula in a 5-year-old child. Due to the different parameters of the bone used, it is not possible to make comparative assessments with respect to the human thigh model using a pig femur. Furthermore, in this study [13], the authors used bones that were not embedded in ballistic gel. Prior work has evaluated femoral shootings with high impact energy projectiles [8–10] however, in these studies, 0.25 inch (6.35 mm) and 0.406 inch (10.03 mm) diameter chromium steel bullets were fired into fixed human femurs not coated with ballistic gelatin. Hence, these studies cannot be contrasted with our observations, regardless of their interesting results. Wightman et al. [11] described lead airgun pellet damage to bovine femoral shaft fragments embedded in small gelatin blocks using pneumatic devices with an exit energy of 16.3J. In Wightman’s study, the pellets did no damage to the bone surface, they were only deformed in contact with the bone.

**Fig 6:**
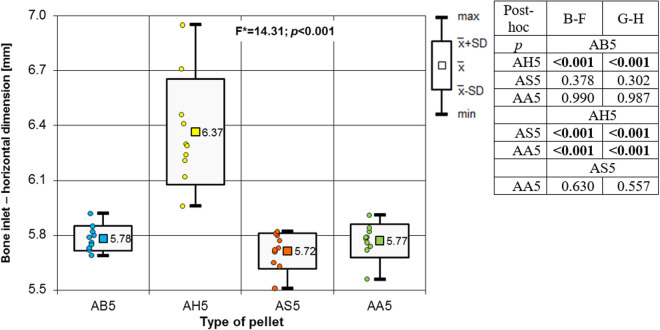
Bone entrance wound – horizontal dimension depending of the type of pellet (result of one way Anova with Brown-Forsythe correction and results of post-hoc tests: Brown-Forsythe [B-F] and Games-Howell [G-H]).

**Fig 7:**
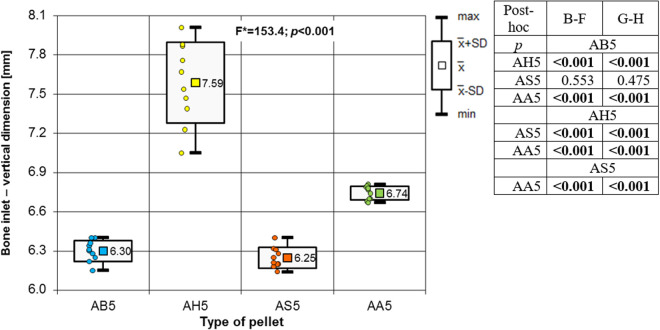
Bone entrance wound – vertical dimension depending of the type of pellet (result of one way Anova with Brown-Forsythe correction and results of post-hoc tests: Brown-Forsythe [B-F] and Games-Howell [G-H]).

**Fig 8:**
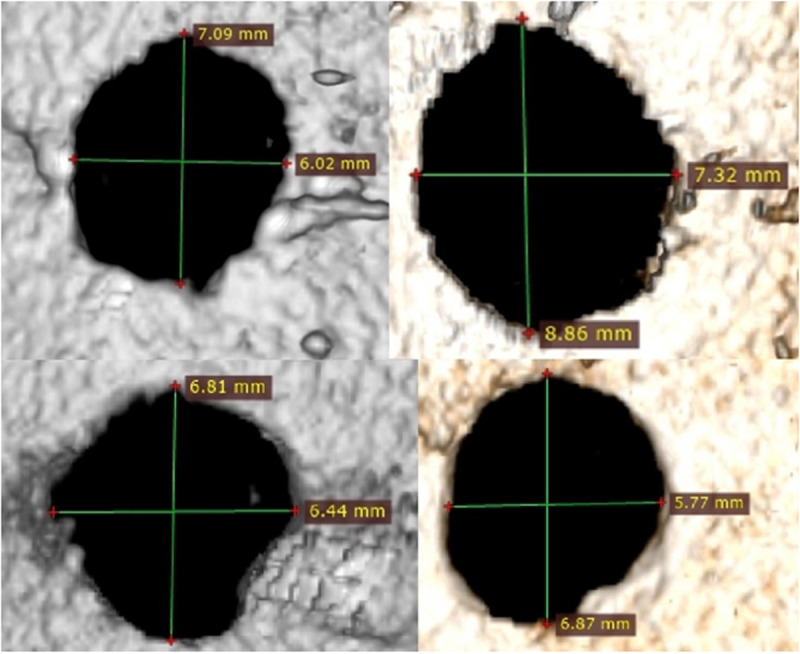
Bone entrance wound damage in 3D CT reconstruction. From upper left, clockwise – H&N Baracuda, HollowPoint, Apollo, Spitzkugel.

**Fig 9:**
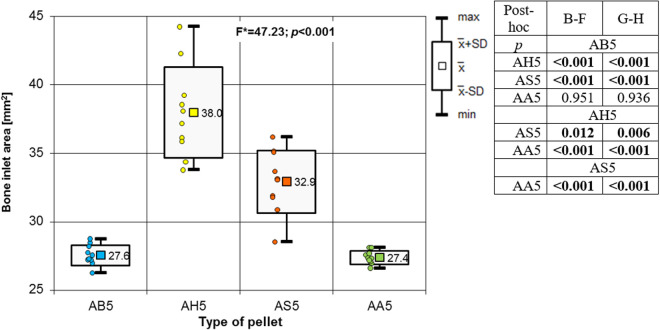
Bone entrance wound area depending of the type of pellet (result of one way Anova with Brown-Forsythe correction and results of post-hoc tests: Brown-Forsythe [B-F] and Games-Howell [G-H]).

**Fig 10:**
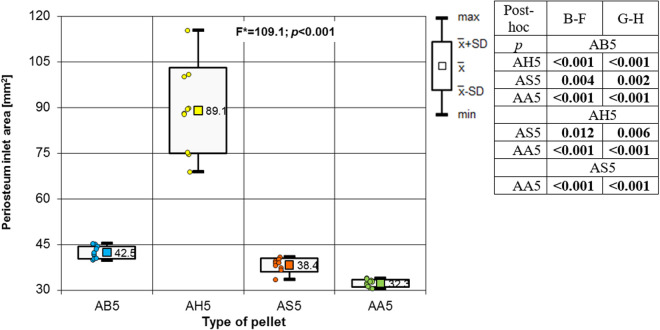
Periosteum entrance wound area depending of the type of pellet (result of one way Anova with Brown-Forsythe correction and results of post-hoc tests: Brown-Forsythe [B-F] and Games-Howell [G-H]).

Analysing the results of the present study, the bone entry lesions tended to be oval in shape, with a vertical dimension slightly larger than the horizontal (Fig 6, Fig 7, Fig 8.). This was true for all the airgun pellets used. This correlation is most likely due to the orientation of osteons in the cortex of the femoral shaft, already described by Koch [23], which run parallel to the long axis of the shaft. When striking the bone, the projectile creates an oval entry hole, with the longer dimension in the long axis of the shaft, which is surrounded by irregularly torn periosteum. The greatest variation in the size of the entry damage in the bone and periosteum was found in the H&N HollowPoint shot (Fig 9, Fig 10.). When analysing the area of entrance damage in bone, statistically significant differences were not shown for the H&N Baracuda and H&N Excite Apollo pellets (Fig 9.). Entrance holes with the largest areas were formed by H&N Hollow Point pellets and the smallest by H&N Apollo and Baracuda pellets (Fig 9.). For periosteal entrance wound damage area, statistically significant differences were shown in all pairs except H&N Baracuda and H&N Spitzkugel (Fig 10.). Although H&N Barracuda is lead pellet, it is very heavy and presents a smaller cross-sectional area than the other projectiles tested, thus creating small resistance when interacting with the bone. Again, the largest damage area was produced by the H&N Hollow Point pellets, and the smallest by the H&N Excite Apollo pellets. For the RA ratio, which is the ratio of periosteal entry damage area to bone entry hole area, all comparisons except the H&N Spitzkugel and H&N Excite Apollo pair showed statistically significant differences. The H&N Hollow Point pellets had the highest RA, while the H&N Spitzkugel and H&N Apollo airgun pellets had the lowest (Fig 10.). Depending on the type, 5.5 mm calibre airgun pellets caused bone and periosteal damage of varying size, morphology and nature.

The most extensive damage within the entry holes was caused by shots with H&N Hollow Point airgun pellets (Fig 6,7,9–13.). A similar situation was observed for damage to the periosteum. Also, the RA coefficient (ratio of the area of periosteal inlet damage to the area of the bone inlet hole) shows the highest values for this pellet (Fig 10.), which is determined by the characteristics of the pellet design. It should be noted that this pellet generated the most damage despite having the lowest impact energy among the pellets used in the tests (Table 3.), which was due to its lowest weight (Table 2). The shape of the H&N Hollow Point pellet, in particular its flattened head with a central recess at the top, causes this pellet to deform more when hitting a hard obstacle in comparison with the other airgun pellets used in the tests. Upon hitting the target, an airgun pellet of such a shape loses its velocity very quickly and deforms. Its deformed head creates a larger hole in the bone than is observed with other airgun pellets, while the flattened calyx damages the periosteum over a much larger area than other pellets. It was also observed, that this airgun pellet produced the biggest number of ricochets (Table 4). The H&N Baracuda and H&N Excite Apollo pellets caused longitudinally oriented fracture lines to propagate from the entry hole (Fig 14, Fig 15.), whereas H&N Spitzkugel did not (Fig 16). The direction of the fracture fissures is probably due to the aforementioned architecture of the femoral shaft. Completely different characteristics from lead pellets are presented by H&N Excite Apollo. It is a lead-free pellet with a teflon sabot. It does not deform upon striking the soft tissue simulant or the bone, leaving a teflon collar on the outside of the bone. The bone holes and damage to the periosteum are the smallest of the four 5.5 mm calibre pellets used in the study. The RA coefficient for the H&N Excite Apollo airgun pellet is very similar to the H&N Spitzkugel pellet, which is a lead pellet with strictly penetration properties (Fig 17.). In testing, these airgun pellets demonstrated very similar velocities and impact energies (Table 5, Table 3). The RA factor for the H&N Excite Apollo pellet is lower than the RA factor for the blunt-headed, rounded-headed, heavy H&N Baracuda pellet. Probable explanation is that H&N Baracuda generates more resistance when hitting a hard target showing a greater tendency to deform than H&N Excite Apollo and H&N Spitzkugel. The H&N Baracuda, the heaviest of the 5.5 mm calibre pellets, is the only one to repetitively perforate through the bone shaft. In contrast, long bone shaft fractures resulting from powder-propelled projectiles involve more extensive fracture patterns, often propagating through and around the shaft [24,25]. The observation of more extensive damage caused by the H&N Baracuda pellet is most likely due to the considerable weight of this airgun pellet (the largest of the pellets used in the study) and the slower dissipation of extensive energy after contact with the target, leading to a higher impact energy when the pellet strikes the bone surface. The closest firearm analogue for 5,5 mm airgun pellets is.22 rimfire ammunition, as calibre is almost the same, but weight of bullets is bigger (from 29 grain upwards) and their kinetic energy is often significantly higher than 50J, even for subsonic ammunition [26,27].

**Table 3:** Impact energy values of 5.5 mm calibre airgun pellets measured at a distance of 10m in Joules [J].

Airgun Pellet	Mean	SD	Max	Min
H&N Baracuda	44,01	0,45	44,40	43,29
H&N Hollow Point	27,10	0,31	27,35	26,66
H&N Spitzkugel	35,23	0,24	34,72	34,11
H&N Excite Apollo	36,57	0,14	36,48	36,14

**Table 4:** Extent of bone damage depending on the type of airgun pellets when shooting into the anterior surface of the pork femur shaft with 5.5 mm calibre pellets.

Airgun Pellet	Penetration of anterior surface of the shaft	Ricochet	Fracture of anterior surface of the shaft	Fracture propagation to lateral surface of the shaft	Fracture propagation to posterior surface of the shaft	Complete perforation of the shaft
AB5	10/10	0	10/10	10/10	10/10	10/10
AH5	10/16	6	7/10	0/10	0/10	0/10
AS5	10/10	0	6/10	0/10	0/10	0/10
AA5	10/10	0	8/10	0/10	1/10	0/10

**Table 5:** Velocities of 5.5 mm calibre airgun pellets measured at a distance of 10m in [m/s].

Airgun Pellet	Mean	SD	Max	Min
H&N Baracuda	253,47	1,29	254,72	251,40
H&N Hollow Point	257,09	1,35	259,23	255,00
H&N Spitzkugel	260,43	0,81	262,11	255,94
H&N Excite Apollo	278,94	0,46	280,12	277,30

**Fig 11:**
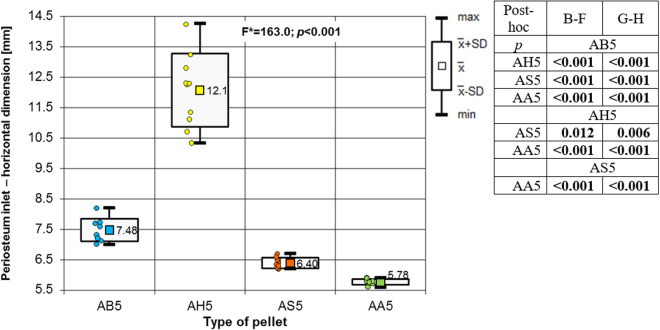
Periosteum entrance wound – horizontal dimension depending of the type of pellet (result of one way Anova with Brown-Forsythe correction and results of post-hoc tests: Brown-Forsythe [B-F] and Games-Howell [G-H]).

**Fig 12:**
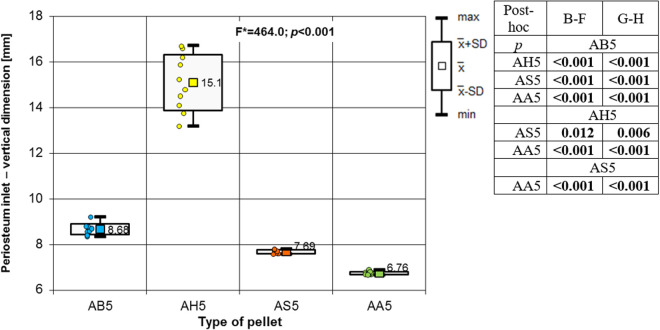
Periosteum entrance wound – vertical dimension depending of the type of pellet (result of one way Anova with Brown-Forsythe correction and results of post-hoc tests: Brown-Forsythe [B-F] and Games-Howell [G-H]).

**Fig 13:**
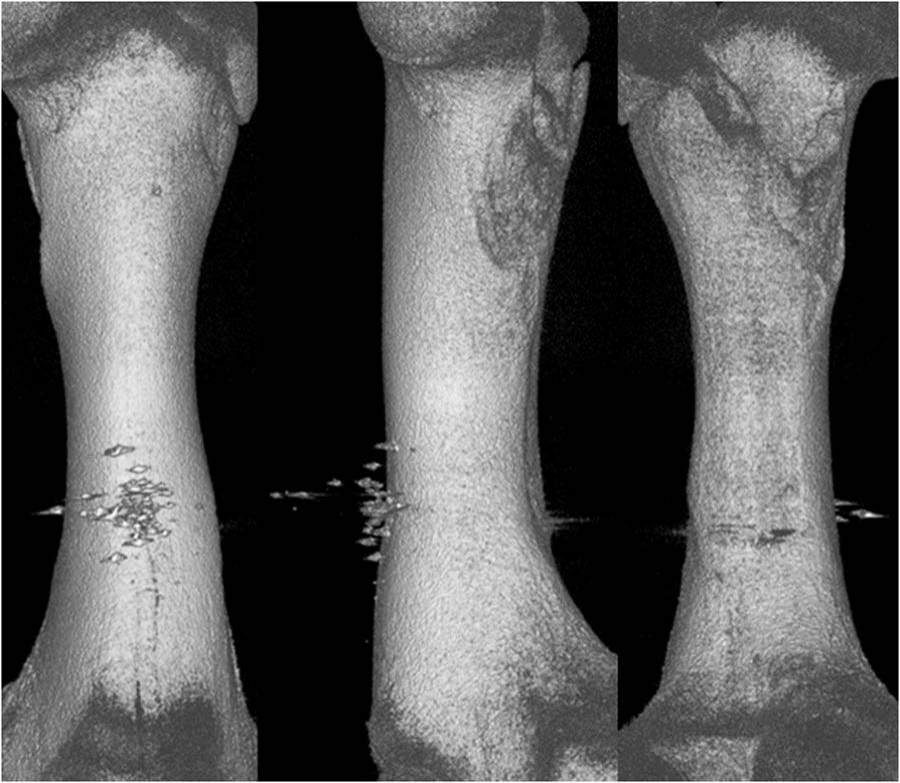
H&N HollowPoint cal. 5.5 mm. 3D CT gunshot damage reconstruction. From the left: anterior, lateral and posterior shaft surfaces.

**Fig 14:**
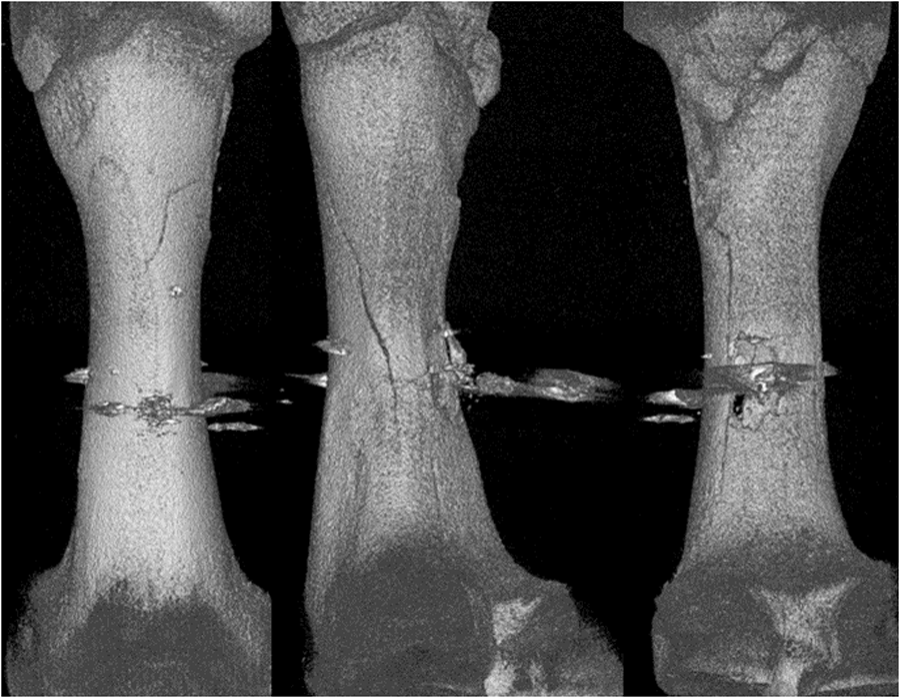
H&N Baracuda cal. 5.5 mm. 3D CT gunshot damage reconstruction. From the left: anterior, lateral and posterior shaft surfaces.

**Fig 15:**
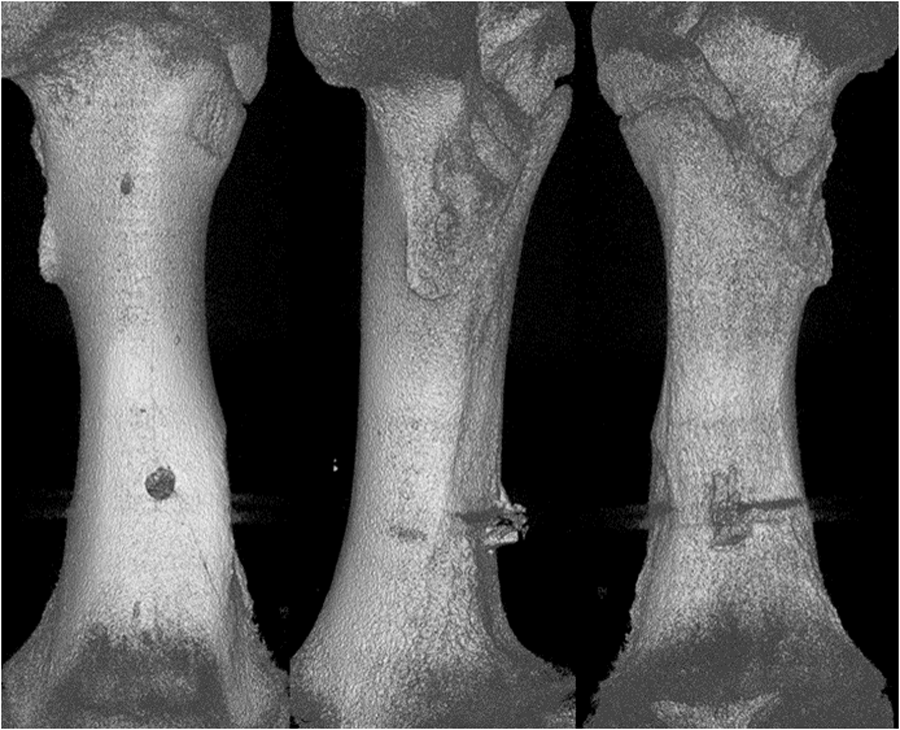
H&N Excite Apollo cal. 5.5 mm. 3D CT gunshot damage reconstruction. From the left: anterior, lateral and posterior shaft surfaces.

**Fig 16:**
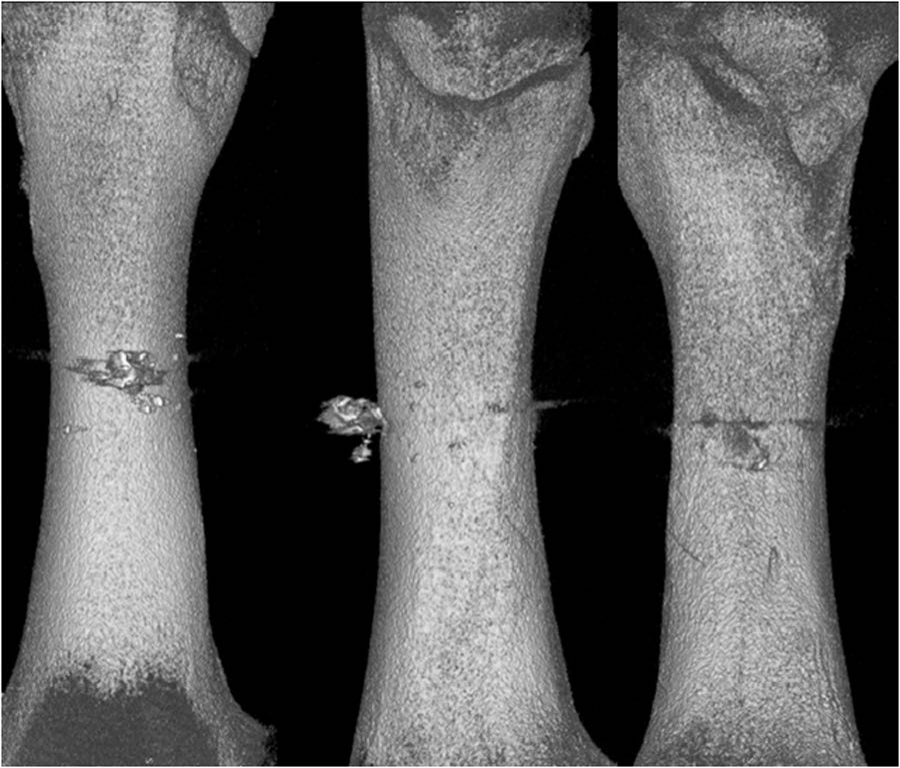
H&N Spitzkugel cal. 5.5 mm. 3D CT gunshot damage reconstruction. From the left: anterior, lateral and posterior shaft surfaces.

**Fig 17:**
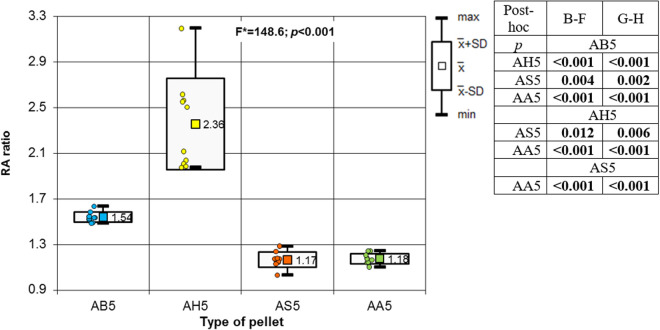
RA ratio depending of the type of pellet (result of one way Anova with Brown-Forsythe correction and results of post-hoc tests: Brown-Forsythe [B-F] and Games-Howell [G-H]).

There are some works describing gunshot damage inflicted with firearms. Study of Schwab N. et al. [28] describes behaviour of Synbone (synthetic bone equivalent) in comparison to deer femur. In general, damage inflicted is similar to our results regarding H&N Baracuda pellet, but because the authors used 9 mm Parabellum ammunition, fired with initial velocity of 360 m/s, impact kinetic energy was significantly, approximately 8 times, greater than in our study. Thus, the results were impossible to compare to our study.

A strength of this study is that the four projectiles tested were of the same calibre and similar muzzle velocity. However, the projectiles differed from each other in mass, material, and nose design (round nose, hollow point, pointed, etc.). Therefore, the data do not support analysis of the relative contributions of impact energy versus rate of energy transfer, which has been useful in studies of powder-propelled bullet injury potential.

Further studies in this area are planned. We plan to perform another test with high-velocity FAC air rifles, focusing on types of newly available airgun pellets. We also plan to perform research on how shaft wall thickness of long bones is related to the extent of damage. The overall goal of this research endeavor is to understand injury potential of airguns using analyses that have proven useful for evaluating injury potential of powder-based firearms.

## Conclusions

1. The extent of gunshot damage to the bone and periosteum of the anterior surface of the femoral shaft is variable and depends on the type of airgun pellet used to inflict the damage.
2. Hollow point pellets caused bone and periosteum damage of the greatest magnitude, while damage of the least extent was generated by the solid tip, unleaded pellets.
3. Solid tip, unleaded pellets generate the entry damage to the bone and periosteum of smaller extent than those generated by lead pellet hit.
4. Bone and periosteal injuries caused by hollow point pellets are the most extensive out of all the pellets used in this study.

## Supporting information

S1 Data (XLSX)
